# *In Vitro* Evaluation of Bioactivity of Chemically Deposited Hydroxyapatite on Polyether Ether Ketone

**DOI:** 10.1155/2015/475435

**Published:** 2015-03-09

**Authors:** D. Almasi, S. Izman, M. Sadeghi, N. Iqbal, F. Roozbahani, G. Krishnamurithy, T. Kamarul, M. R. Abdul Kadir

**Affiliations:** ^1^Department of Manufacturing and Industrial Engineering, Faculty of Mechanical Engineering, Universiti Teknologi Malaysia, 81310 Skudai, Johor, Malaysia; ^2^Medical Devices & Technology Group (MEDITEG), Faculty of Bioscience and Medical Engineering, Universiti Teknologi Malaysia, 81310 Skudai, Johor, Malaysia; ^3^Faculty of Chemical Engineering, Universiti Teknologi Malaysia, 81310 Johor Bahru, Johor, Malaysia; ^4^Faculty of Bioscience and Medical Engineering, Universiti Teknologi Malaysia, 81310 Skudai, Johor, Malaysia; ^5^Institute of Translational Medicine, University of Liverpool, Sherrington Building, Liverpool L69 3GE, UK; ^6^Department of Orthopaedic Surgery, NOCERAL, Faculty of Medicine, University of Malaya, 50603 Kuala Lumpur, Malaysia

## Abstract

Polyether ether ketone (PEEK) is considered the best alternative material for titanium for spinal fusion cage implants due to its low elasticity modulus and radiolucent property. The main problem of PEEK is its bioinert properties. Coating with hydroxyapatite (HA) showed very good improvement in bioactivity of the PEEK implants. However the existing methods for deposition of HA have some disadvantages and damage the PEEK substrate. In our previous study a new method for deposition of HA on PEEK was presented. In this study cell proliferation of mesenchymal stem cell and apatite formation in simulated body fluid (SBF) tests were conducted to probe the effect of this new method in improvement of the bioactivity of PEEK. The mesenchymal stem cell proliferation result showed better cells proliferation on the treated layer in comparison with untreated PEEK. The apatite formation results showed the growth of the HA on the treated PEEK but there was not any sight of the growth of HA on the untreated PEEK even after 2 weeks. The results showed the new method of the HA deposition improved the bioactivity of the treated PEEK in comparison with the bare PEEK.

## 1. Introduction

Recently PEEK has been considered as an ideal choice for spinal fusion cage due to its low modulus of elasticity, excellent chemical stability, resistance to radiation used in sterilization procedures, transparency to radio waves, compatibility with reinforcing agent, and so forth. Food and Drug Administration of United States (FDA) accepted the carbon-fiber-reinforced PEEK (CFR-PEEK) for spinal fusion cages for human use in 1990s.

There is one big disadvantage of PEEK for orthopedic application, which is its low bioactivity. For enhancement of the bioactivity of PEEK, several methods have been proposed such as plasma surface treatment [1], laser surface modification [2], deposition of the HA coating layer [3], and compounding with bioactive material such as HA and producing bioactive composites [4]. The current commercial method for improving the bioactivity of PEEK is deposition of HA via plasma spraying method which has some disadvantages due to the high temperature which is used during the deposition of the HA coating layer and crystallization of the coated HA [3]. In our previous study the HA crystalline particles were deposited on PEEK and eliminate high temperature in the deposition process [5].

In this study the bioactivity of the HA coated samples via chemically deposition method was evaluated by* in vitro* study. Mesenchymal stem cell proliferation and apatite formation via SBF immersion were used for doing the* in vitro* bioactivity study.

## 2. Experimental Procedures

### 2.1. Sample Preparation

The treated samples were prepared as explained in our previous study [5]. The sulphonation time was chosen as 5 minutes for the samples.

### 2.2. Apatite Formation Study

Bioactivity can be understood as biologically reaction of the tissue to the surface of the materials. Formation of apatite layer from simulated body fluid (SBF) is one of the widely used methods which enables predicting* in vivo* bone bonding tendency of the material [6, 7]. The first SBF was developed by Kokubo and his colleagues in 1987, to simulate the reaction to the material* in vitro* instead of* in vivo* conditions [8]. It was designed to study the bioactivity of materials* in vitro* as a preliminary cheap and easy evaluation.

The samples were immersed in SBF solution and kept in water bath with the fixed temperature of 37°C. The ratio of the surface of the sample to the volume of the SBF solution had a value almost equal to 0.02 cm^−1^ [9]. SEM and EDX analysis were used to show the apatite formation on the surface coated and bare PEEK after immersion in SBF and also for showing the morphology of the attached cells to the samples. Hitachi Tabletop SEM TM-3000 equipment was used for SEM and EDX evaluation.

### 2.3. *In Vitro* Evaluation of HA Coated PEEK Using Mesenchymal Stem Cells

Biocompatibility evaluation was performed using Mesenchymal stem cells (hBMSCs). Mesenchymal stem cells (hBMSCs) were harvested and cultured as described by Krishnamurithy et al. [10]. The 2nd passage was used for samples cell adhesion experiment.

Cell experiments were carried out on the control bare PEEK discs and the surface treated PEEK discs, which were 12 mm in diameter and 3 mm in thickness. Prior to doing the experiments, all samples were sterilized under UV irradiation for 1 hr.

#### 2.3.1. Cell Attachment

The adhesion of Mesenchymal stem cells (hBMSCs) on disc samples was assessed in individual 6-well culture plate. The cells were seeded on the samples at a density of 1 × 10^6^ cells/mL. The seeded test samples were incubated with osteogenic medium (Gibco, Invitrogen, USA) at 37°C in a CO_2_ incubator under standard culture conditions. Medium were changed after 1, 5, and 10 days and used for cell viability test.

The disc samples at day 10 were fixed overnight in 4% glutaraldehyde in 0.1 M cacodylate buffer and postfixed for 1 hr in 1% aqueous osmium tetroxide. These samples were processed with three consecutive washing steps in distilled water before being dehydrated through a graded ethanol series (50, 70, 80, 90, 95, and 100%). The samples were subsequently dried overnight at room temperature. Then the samples were sputter coated with gold before being examined using the SEM.

#### 2.3.2. Alamar Blue Assay

Cell viability on HA coated and bare PEEK samples was assessed using the colorimetric indicator Alamar blue assay (Gibco, Invitrogen, USA). The assay was carried out based on the % of Alamar blue (AB) reduction on day 5, and 10. AB was directly added into the DMEM media in all preparations at final concentration of 10% and incubated for 10 hrs. After incubation, 100 *μ*L of medium from each plate was transferred into a 96-well plate. AB added to media without cells served as a negative control. Absorbance in each well was measured at testing wavelength of 570 nm and reference wavelength of 600 nm using a micro plate reader (EPOCH, USA). After the AB, the cell-disc samples were then washed with 1x PBS (phosphate buffered saline 1x: Gibco, Invitrogen, USA) and subsequently incubated in new DMEM medium.

The concentrations were analyzed by SPSS Statistics 22 (IBM, USA) using one-way ANOVA and Tukey's test followed by post hoc LSD, with a significant level set at *P* < 0.05.

## 3. Results and Discussion

**Figure 1:**
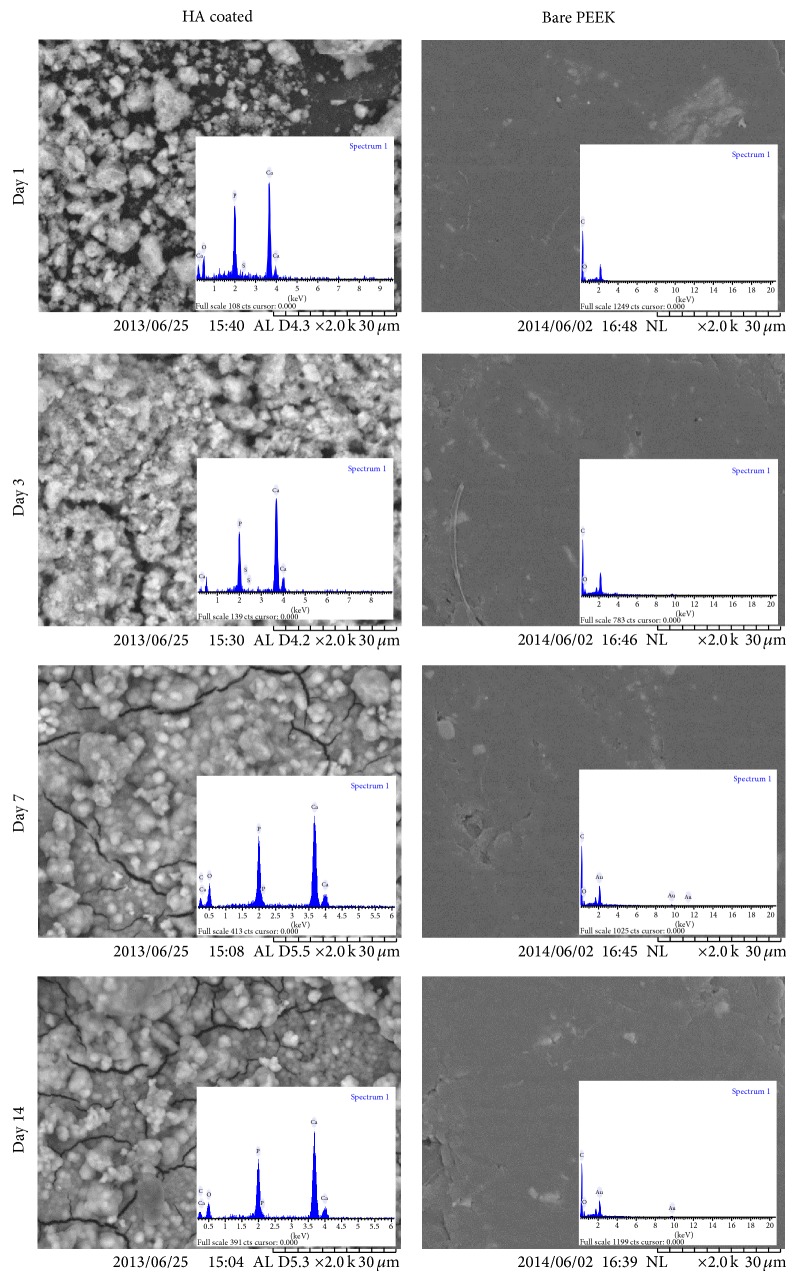
SEM images of the apatite formation on the PEEK samples with and without coating layer.

Figure 1 shows the SEM images and EDX results of the HA coated and bare PEEK samples after immersion in the SBF up to 14 days. Apatite formation is completely visible and confirmed the bioactivity of the coated PEEK. After 7 days the whole surface of the HA coated samples were covered by spherical apatite layer. The EDX analysis for the samples after 1 and 3 days immersion in SBF still showed the sulfur element which belongs to the surface layer of the coated samples, but after 7 days due to the complete covering of the apatite layer the sulfur element does not exist in the surface. There was no apatite formation observed on the surface of the bare PEEK samples after 2-week immersion in SBF. The results showed the coated layer PEEK has better apatite formation ability which can have better interaction with the surrounding bone in the body.

**Figure 2:**
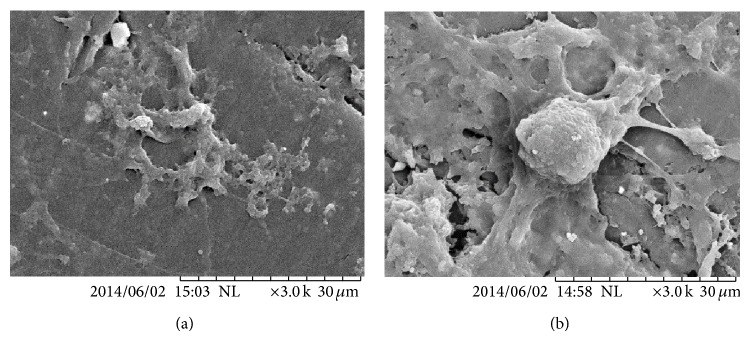
SEM image of the morphology of the attached cell on the (a) bare PEEK, (b) treated layer.

The effect of the surface treatment on the biocompatibility of the samples was investigated by comparing the cell behavior.  Figure 2 shows the morphology of mesenchymal stem cells (hBMSCs) on the samples after culturing for 10 day. From SEM images, it can be seen that the cells attached and spread on both surfaces indicating that all the surfaces were able to support cell growth and metabolic activity of hBMSCs. Samples showing wider coverage of lamellipodial and filopodial extension indicates the improvement in biocompatibility due to surface treatment. The formation of bridges among the cells indicated that the surface may be favourable for the cells to prolong active metabolism for viability and proliferation [10]. Moreover, the architecture around the cells mimicked extracellular matrix (ECM) and formed a continuous layer of fusiform over the surface of the HA treated PEEK substrate.

**Figure 3:**
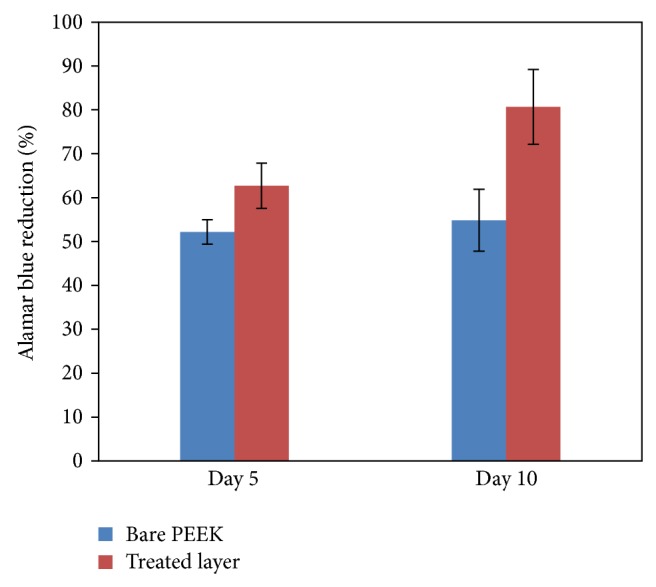
hBMSC proliferation on bare PEEK and PEEK treated layer (Alamar blue assay).

The percentage of reduction of AB was monitored on days 5 and 10 (Figure 3). The data obtained showed a significant difference in cell proliferation on HA treated PEEK substrate when compared to HA untreated PEEK substrate on day 5 ((*P* < 0.05) and 10 (*P* < 0.01)). The proliferation rate of cells seeded on the HA treated PEEK substrate was also found to be significantly higher on Day 10, when compared with that of day 5 (*P* < 0.01). However, there was no significant difference in the cell proliferation rate between days 5 and 10 in HA untreated PEEK substrate (*P* > 0.05). A previous study also reported similar outcome where the proliferation rate of osteoblast seeded on the HA coated PEEK was increased, when compared with a bare PEEK substrate between days 1 and 3. This resultant outcome suggested that the surface of HA treated PEEK substrate is favourable in terms of cell affinity [11].

## 4. Conclusions

*In vitro* study via simulated body fluid was conducted to compare the bioactivity of bare PEEK with coated PEEK with HA particles via chemical deposition. HA crystals did not grow on the immersed bare PEEK in SBF, which showed the bioinert property of PEEK. For the coated PEEK with HA particle,* in vitro* study via SBF immersion showed the growth of HA crystals from the first day of immersion in SBF and the HA crystals growth each day which show the bioactivity property of the coated PEEK.* In vitro* study via hBMSCs cell attachment and proliferation confirms the improvement of the bioactivity of the coated PEEK in comparison with the bare PEEK, although the new method for HA deposition needs more studying such as* in vivo* study before using in human body.

## Highlights

- The bioactivity of the modified PEEK was studied via mesenchymal stem cell.
- The apatite formation of the modified PEEK was studied via SBF immersion.
- *In vitro* study revealed increase in bioactivity of modified PEEK.
